# Nrf2 protects human alveolar epithelial cells against injury induced by influenza A virus

**DOI:** 10.1186/1465-9921-13-43

**Published:** 2012-06-06

**Authors:** Beata Kosmider, Elise M Messier, William J Janssen, Piruz Nahreini, Jieru Wang, Kevan L Hartshorn, Robert J Mason

**Affiliations:** 1 Department of Medicine, National Jewish Health, 1400 Jackson Street, Denver, CO, 80206, USA; 2 Department of Hematology/Oncology, Boston University School of Medicine, Boston, MA, 02118, USA

**Keywords:** Human alveolar epithelial cells, Alveolar macrophages, Influenza A virus, Nrf2, Apoptosis, Efferocytosis

## Abstract

**Background:**

Influenza A virus (IAV) infection primarily targets respiratory epithelial cells and produces clinical outcomes ranging from mild upper respiratory infection to severe pneumonia. Recent studies have shown the importance of lung antioxidant defense systems against injury by IAV. Nuclear factor-erythroid 2 related factor 2 (Nrf2) activates the majority of antioxidant genes.

**Methods:**

Alveolar type II (ATII) cells and alveolar macrophages (AM) were isolated from human lungs not suitable for transplantation and donated for medical research. In some studies ATII cells were transdifferentiated to alveolar type I-like (ATI-like) cells. Alveolar epithelial cells were infected with A/PR/8/34 (PR8) virus. We analyzed PR8 virus production, influenza A nucleoprotein levels, ROS generation and expression of antiviral genes. Immunocytofluorescence was used to determine Nrf2 translocation and western blotting to detect Nrf2, HO-1 and caspase 1 and 3 cleavage. We also analyzed ingestion of PR8 virus infected apoptotic ATII cells by AM, cytokine levels by ELISA, glutathione levels, necrosis and apoptosis by TUNEL assay. Moreover, we determined the critical importance of Nrf2 using adenovirus Nrf2 (AdNrf2) or Nrf2 siRNA to overexpress or knockdown Nrf2, respectively.

**Results:**

We found that IAV induced oxidative stress, cytotoxicity and apoptosis in ATI-like and ATII cells. We also found that AM can ingest PR8 virus-induced apoptotic ATII cells (efferocytosis) but not viable cells, whereas ATII cells did not ingest these apoptotic cells. PR8 virus increased ROS production, Nrf2, HO-1, Mx1 and OAS1 expression and Nrf2 translocation to the nucleus. Nrf2 knockdown with siRNA sensitized ATI-like cells and ATII cells to injury induced by IAV and overexpression of Nrf2 with AdNrf2 protected these cells. Furthermore, Nrf2 overexpression followed by infection with PR8 virus decreased virus replication, influenza A nucleoprotein expression, antiviral response and oxidative stress. However, AdNrf2 did not increase IFN-λ1 (IL-29) levels.

**Conclusions:**

Our results indicate that IAV induces alveolar epithelial injury and that Nrf2 protects these cells from the cytopathic effects of IAV likely by increasing the expression of antioxidant genes. Identifying the pathways involved in protecting cells from injury during influenza infection may be particularly important for developing new therapeutic strategies.

## Background

Influenza A virus (IAV) targets the lung epithelial cells for infection and produces clinical outcomes ranging from a mild upper respiratory infection to severe pneumonia [1,2]. Influenza viruses cause oxidative stress and acute respiratory inflammation [3,4]. Recent studies have focused on the role of lung antioxidant defense systems against injury induced by this virus because they likely play a role in virus-associated inflammation, viral susceptibility and immune clearance [5,6]. It has been shown that antioxidant compounds inhibit influenza virus replication and diminish the release of inflammatory and apoptotic mediators during virus infection [7]. Moreover, the combination of antioxidants with antiviral drugs synergistically reduces the lethal effects of influenza virus infections. This suggests that agents with antiviral and antioxidant activities could be a strategy for the treatment of patients with severe influenza-associated complications [8].

Epithelial cells are the primary site of viral replication for influenza virus. Upon viral entry, IAV inhibits host cell protein synthesis and initiates fast and efficient viral replication. The end result of this process is host cell apoptosis and cytotoxicity [9]. Apparently, neighboring cells sense the presence of apoptotic cells and actively extrude them from the monolayer [10-12].

Nuclear factor-erythroid 2 related factor 2 (Nrf2) is a member of the family of cap’n’collar basic leucine zipper transcription factors [6] and, although it is ubiquitously expressed throughout the lung, it is found predominantly in the epithelium and alveolar macrophages (AM) [13]. The activation of the majority of antioxidant and defense genes are regulated by Nrf2 through binding to antioxidant response elements (AREs) [6]. It has been recently reported that the antioxidant pathway controlled by Nrf2 is pivotal for protection against the development of influenza virus-induced pulmonary inflammation and lung injury in mice *in vivo* under oxidative conditions [6]. Nrf2 also plays a key role in host defense against respiratory syncytial virus (RSV) *in vivo*[14], and RSV infection induces down-regulation of airway antioxidant systems in mice [15]. Moreover, Cho et al. reported that Nrf2 has antiviral activity in murine models of RSV [14], and Nrf2 activation by epigallocatechin gallate decreased viral replication in response to influenza A/Bangkok/1/79 infection in human nasal epithelial cells [5]. These results suggest that attenuation of oxidative stress, inflammation and apoptosis may lessen influenza virus-induced lung injury and exacerbation of existing respiratory diseases.

Children are particularly susceptible to influenza, they account for numerous outpatient visits, and have a central role for spreading infection within the community [16,17]. It has also been reported that neonatal mice are more susceptible to A/PR/8/34 (PR8) virus than adult mice [18]. Therefore in this study, we chose to use alveolar type II (ATII) cells from pediatric lung donors to focus on the response of lung cells isolated from children. We have recently reported the effects of influenza on alveolar epithelial cells from adults [19].

In the current study, we used human primary alveolar type I-like (ATI-like) and ATII cells. Alveolar type I (ATI) cells are large flat cells that cover ~ 95% of the alveolar surface and through which gas exchange takes place. ATII cells make and secrete pulmonary surfactant, and they proliferate to restore the epithelium after damage to the more sensitive ATI cells [20]. Human ATI cells have not been isolated and cultured. We chose to use ATI-like cells, which are type II cells cultured to transdifferentiate into a ATI cell phenotype *in vitro*[20-22]. We analyzed clearance of PR8 virus-induced apoptotic cells by human primary AM and ATII cells. Furthermore, to improve our knowledge on pathways involved in lessening the cellular injury associated with influenza infection, we focused on the role of Nrf2 in human primary alveolar epithelial cells infected with IAV. This is the first study on the effect of IAV on ATI-like cells and to our knowledge there is no study of the role of Nrf2 in influenza A virus infection in cells obtained from children. Our hypothesis is that Nrf2 protects alveolar cells against injury induced by PR8 virus by activation of antioxidant defense genes and decreasing oxidative stress and viral replication. To test this hypothesis we used adenovirus Nrf2 (AdNrf2) and Nrf2 siRNA strategies to study the effect of Nrf2 overexpression and knockdown, respectively in these cells infected by PR8 virus.

## Methods

### Donor information

Deidentified human lungs not suitable for transplantation were donated for medical research from the National Disease Research Interchange (Philadelphia, PA) and the International Institute for the Advancement of Medicine (Edison, NJ). For this study we selected non-smoker lung donors (N = 6, 2-18 years old). The Committee for the Protection of Human Subjects at National Jewish Health approved this research. To our knowledge this is the first report using alveolar epithelial cells from children to study the effect of IAV.

### Isolation and culture of ATII cells, ATI-like cells and AM

The ATII cell isolation method has been published previously [23,24]. Briefly, the right middle lobe was perfused and lavaged, and then instilled with elastase (Roche Diagnostics, Indianapolis, IN). Subsequently, the lung was minced and the cells were filtrated and purified by centrifugation on a density gradient made of Optiprep (Accurate Chemical Scientific Corp., Westbury, NY) and by negative selection with CD14-coated magnetic beads (Dynal Biotech ASA, Oslo, Norway) and binding to IgG-coated (Sigma Chemicals Inc., St. Louis, MO) dishes. The purity of ATII cells was ~80% before plating and over 95% after adherence in culture [20].

The isolated ATII cells were cultured as we described previously [19,23]. Briefly, they were resuspended in DMEM supplemented with 10% fetal bovine serum (FBS), 2 mM glutamine, 100 μg/ml streptomycin, 100 U/ml penicillin (all from Thermo Scientific, Franklin, MA), 2.5 μg/ml amphotericin B (Mediatech Inc., Manassas, VA) and 10 μg/ml gentamicin (Sigma Chemicals Inc., St. Louis, MO). To maintain their differentiated state, ATII cells were plated for 2 d with 10% FBS on millicell inserts (Millipore Corp., Bedford, MA) coated with a mixture of 20% Engelbreth-Holm-Swarm tumor matrix (BD Biosciences, San Jose, CA) and 80% rat-tail collagen (RTC) in DMEM with additives as mentioned above and then cultured for 2 d with 1% charcoal-stripped FBS along with 10 ng/ml keratinocyte growth factor (KGF, R&D Systems Inc., Minneapolis, MN), and for an additional 2 d with 10 ng/ml KGF, 0.1 mM isobutylmethylxanthine, 0.1 mM 8-Br-cAMP, and 10 nM dexamethasone (all from Sigma Chemicals Inc., St. Louis, MO) [19].

To transdifferentiate ATII cells into ATI-like cells, ATII cells were plated on RTC-coated plates or glass coverslips in DMEM with 10% FBS for 2 d and then cultured in DMEM with 5% FBS for 4 d [20] in addition to glutamine, amphotericin B, streptomycin, penicillin, and gentamicin as mentioned above.

AM were isolated as we previously described [25]. Briefly, the lung was lavaged with HEPES-buffered saline and 2 mM EDTA and the lavage fluid was centrifuged at 4°C for 10 min. The resulting pellet was resuspended and plated in DMEM supplemented with 10% FBS in addition to glutamine, amphotericin B, streptomycin, penicillin, and gentamicin as mentioned above. After 24 h, AM were cultured for 2 d in DMEM with 5% FBS.

### Infection with PR8 virus, AdNrf2 and AdGFP

The H1N1 strain A/PR/8/34 (PR8) (used interchangeably with IAV throughout the manuscript) was an original gift from Dr. J. Abramson (Bowman Gray School of Medicine, Winston-Salem, NC). PR8 virus was grown in 10-day-old chicken eggs and virus-containing allantoic fluid was processed as previously reported [26]. ATI-like and ATII cells were infected with PR8 virus as we described [25]. Briefly, cells were inoculated with PBS or PR8 virus at a MOI of 0.05, 0.5 and 1 pfu/cell. After 1 h cells were washed twice with DMEM and incubated for 24 h or 48 h.

Adenovirus Nrf2 (AdNrf2) with green fluorescent protein (GFP) and adenovirus GFP (AdGFP) were obtained from Dr. Timothy H. Murphy [27]. For adenovirus infection in ATI-like or ATII cells we used virus diluted to a MOI of 200 pfu/cell in PBS. Cells were allowed to express transgenes for 24 h before usage. All infected cell cultures were examined for adequate infection efficiency as assessed by GFP fluorescence (88% for ATI-like cells and 85% for ATII cells) and by western blotting for Nrf2.

### Cell transfection with Nrf2 siRNA

Nrf2 siRNA duplex showing maximum knockdown in A549 cells (sense: 5’ CAGCAGAACUGUACCUGUUUU 3’; antisense: 3’ UUGUCGUCUUGACAUGGACAA 5’) [28] was purchased from Dharmacon Research, Inc (Lafayette, CO). To confirm the specificity of the inhibition, the control, nontargeting (NT) siRNA was used as negative control (sense: 5’ UAGCGACUAAACACAUCAAUU 3’; antisense 3’ UUAUCGCUGAUUUGUGUAGUU 5’) [28]. Cells were transfected with 100 nmol of siRNA duplexes by using GenomONE HVJ Envelope Vector Kit (Cosmo Bio CO. Ltd. Carlsbad, CA) according to the manufacturer’s instructions. After 24 h, cells were infected with PR8 virus as described above. Knockdown of the target gene was quantified by western blotting with GAPDH for normalization.

### Immunocytofluorescence

To detect viral antigen, ATI-like cells were fixed with methanol and blocked with 3% normal donkey serum (Jackson ImmunoResearch; West Grove, PA) in PBS. The cells were incubated with an antibody specific to influenza A nucleoprotein (Millipore Corp., Billerica, MA) and anti-cytokeratin MFN116 (Dako, Carpinteria, CA). The secondary antibody, Alexa Fluor 594 IgG (Invitrogen Corp., Carlsbad, CA) and Alexa Fluor 488 IgG were applied for 1 h. Cells were mounted with Vectashield medium containing DAPI (Vector Laboratories, Burlingame, CA).

The same protocol was used to detect Nrf2 translocation in ATI-like cells, ATII cells and AM infected with PR8 virus. Cells were incubated with rabbit anti-Nrf2 antibody (Santa Cruz Biotechnology Inc., Santa Cruz, CA) and subsequently with Alexa Fluor 594 anti-rabbit IgG.

To detect oxidative stress induced by PR8 virus we applied rabbit anti-4-hydroxynonenal (4-HNE) antibody (Abcam, Cambridge, MA). 4-HNE is a product of lipid peroxidation and hence a marker of oxidative stress. We used Alexa Fluor 594 anti-rabbit IgG as described above.

### Efferocytosis

Floating ATII cells were collected from control cells and cells infected at a MOI of 1 pfu/cell PR8 virus for 48 h. The latter ones contained around 70% apoptotic cells as detected by ethidium bromide and acridine orange double staining (data not shown). This method is more sensitive than TUNEL (TdT-mediated dUTP Nick-End Labeling) assay and allows distinguishing early and late apoptotic cells from necrotic or alive cells [23]. To study efferocytosis we used PKH26 Red Fluorescent Cell Linker Kit and PKH2 Green Fluorescent Phagocytic Cell Linker Kit (both from Sigma, St. Louis, MO) according to the manufacturer’s instructions. PKH2 labeled AM were incubated with PKH26 labeled floating ATII cells (ratio 1:10) for 3 h. Cells were mounted with Vectashield medium containing DAPI. A minimum 200 AM were counted. The phagocytic index was calculated using the following formula: ((number of apoptotic bodies)/(200 total macrophages)) x 100 [29].

### Detection of necrotic cells using propidium iodide and Hoechst 33342 double staining

To distinguish between live and necrotic cells 10 mg/ml Hoechst 33342 and 1 mg/ml propidium iodide (both from Sigma Chemicals Inc., St. Louis, MO) were used for a double staining. Three hundred cells were analyzed in each of three independent experiments [30].

### Western blotting

Expression of proteins was measured by western blotting according to the protocol described previously [23]. Protein loading was normalized to GAPDH. We used mouse anti-GAPDH, rabbit anti-caspase 3 and rabbit anti-caspase 1 (all from Abcam, Cambridge, MA), mouse anti-HO-1 (Assay Designs, Ann Arbor, MI), rabbit anti-Nrf2 (Santa Cruz Biotechnology, Santa Cruz, CA) and mouse anti-influenza A nucleoprotein (Millipore, Corp., Bedford, MA). The blots were then developed using an enhanced chemiluminesence (ECL) western blotting kit according to the manufacturer’s instructions (Amersham Pharmacia Biotech, Piscataway, NJ). Images obtained were quantitated using NIH Image 1.62 software.

### Real-time PCR

Total RNA was isolated from cells using the RNeasy Mini kit (Qiagen, Valencia, CA) according to the manufacturer’s recommendations. Taqman qPCR was performed on a CFX C1000 CFX96 Thermocycler (Biorad, Hercules, CA). Probes and cycling condition were optimized in accordance with MIQE guidelines for PCR [31]. Gene expression levels were calculated as a ratio to the expression of the reference gene, GAPDH and data were analyzed using the ΔΔCt method. The probes for Nrf2, HO-1, Mx1 and OAS1 were designed by the manufacturer and purchased from Applied Biosystems (Carlsbad, CA).

### Plaque assay

Plaque assay was performed as we previously described [32]. Briefly, medium from PR8 virus infected ATI-like cells was serially diluted in DMEM and used to inoculate Madin-Darby canine kidney (MDCK) cells. Confluent MDCK cells were infected with PR8-infected ATI-like cell supernatant for 1 h at 37°C. The inoculum was removed and the cells were overlaid with MEM, FBS, antibiotics and SeaKem LE Agarose (Cambrex, Rockland, ME). Plaques were stained after 72 h incubation at 37°C, with the agarose overlay medium containing 6% Neutral Red (Sigma, St. Louis, MO).

### ELISA

IL-8 and IL-29 (IFN-λ1) were measured by ELISA (ELISA Tech., Aurora, CO) in the ATI-like and ATII cell culture supernatant according to the manufacturer's recommendations. We used a MicroQuant microplate spectrophotometer (BioTek Instruments, Winooski, VT) and analyzed with KCjunior Data Analysis Software.

### ROS production

We compared ROS production in ATI-like and ATII cells transfected with Nrf2 siRNA or AdNrf2 followed by infection with PR8 virus at a MOI of 0.5 pfu/cell for 24 h. We used the Amplex Red Hydrogen Peroxide Assay Kit (Invitrogen Corp., Carlsbad, CA) as a quantititative index of ROS generation [33]. Because H_2_O_2_ is one of the most stable forms of ROS, this detection method allows observation of oxidation processes in real time. Amplex Red reacts with hydrogen peroxide in the presence of horseradish peroxidase (HRP) with a 1:1 stoichiometry to form resorufin. Briefly, 50 μl of samples and standards were mixed with 50 μl of 100 μM Amplex Red and 0.2 U/ml HRP solution and incubated for 30 min at room temperature. Absorbance was measured at 560 nm and calculated concentrations were normalized to protein content.

### Glutathione measurement

ATI-like cells and ATII cells were cultured and infected at a MOI of 0.5 pfu/cell PR8 virus for 24 h. Total glutathione (GSH) was analyzed as previously described [34,35]. Briefly, GSH was measured by mixing 100 μl of 1:1 3 U/ml glutathione reductase with 0.67 mg/ml 5,5’-Dithiobis (2-nitrobenzoic acid, DTNB) with sample or standard. The reaction was initiated by the addition of 50 μl of 0.67 mg/ml NADPH (all from Sigma, St. Louis, MA) Absorbance was measured spectrophotometrically at 412 nm and obtained values were normalized to protein content.

### Statistical analysis

One-way ANOVA by GraphPad Prism 4 was used to evaluate statistical differences among experimental groups. A Dunnett’s test was applied and a value of *p* < 0.05 was considered significant. Data are shown here as the mean ± SEM from three independent experiments.

## Results

### PR8 virus infects ATI-like cells

**Figure 1:**
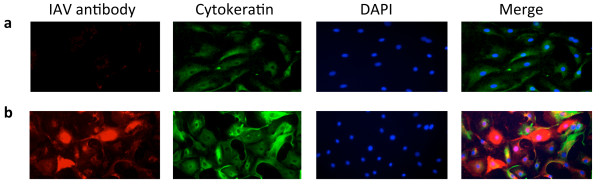
**PR8 virus infects ATI-like cells.** ATI-like cells were cultured in DMEM with 10% FBS for 2 d and then cultured in DMEM with 5% FBS for 4 d. These cells were inoculated with PR8 virus for 1 h at a MOI of 1 pfu/cell and harvested at 48 hpi. Representative picture of positive immunocytofluorescent stain using IAV antibody (red), cytokeratin MFN116 (green) and DAPI (blue): Panel **a** – mock infected cells; Panel **b** – ATI-like cells inoculated with PR8 virus. Representative pictures are from three independent experiments.

We wanted to study the role of Nrf2 and oxidative injury during influenza infection. In this report we focused mostly on ATI-like cells. The alveolar wall is covered primarily by ATI cells and these cells are more sensitive to oxidative injury than ATII cells. Our initial study was simply to document that ATI-like cells could be infected at comparable levels to our previous observations with ATII cells [19]. We used cytokeratin MFN 116 as a marker of ATI-like cells, and we found that PR8 virus readily infected these cells (Figure 1) similar to what we have observed with ATII cells.

### PR8 virus induces apoptosis and cytoxicity in alveolar epithelial cells

IAV induces apoptosis and secondary necrosis *in vitro* because of the lack of phagocytes. We wanted to study the role of Nrf2 in preventing cell injury, therefore, we determined the extent of cytotoxicity of PR8 virus, which can result in apoptosis and/or necrosis. We observed necrosis in cells infected with PR8 virus and a higher percentage of necrotic ATI-like cells than ATII cells (Additional file 1: Figure S 1). We used TUNEL assay to determine whether MOI of 0.05, 0.5 or 1 pfu/cell PR8 virus induces apoptosis after 24 h or 48 h. We observed morphological characteristics of apoptosis (Additional file 2: Figure S 2, Panel I) and a higher percentage of apoptotic cells in the floating cell population than attached ATI-like and ATII cells (Additional file 2: Figure S 2, Panel II). We also found a statistically significant higher percentage of apoptotic ATI-like cells than ATII cells, which suggests that these cells are more sensitive to injury induced by PR8 virus.

**Figure 2:**
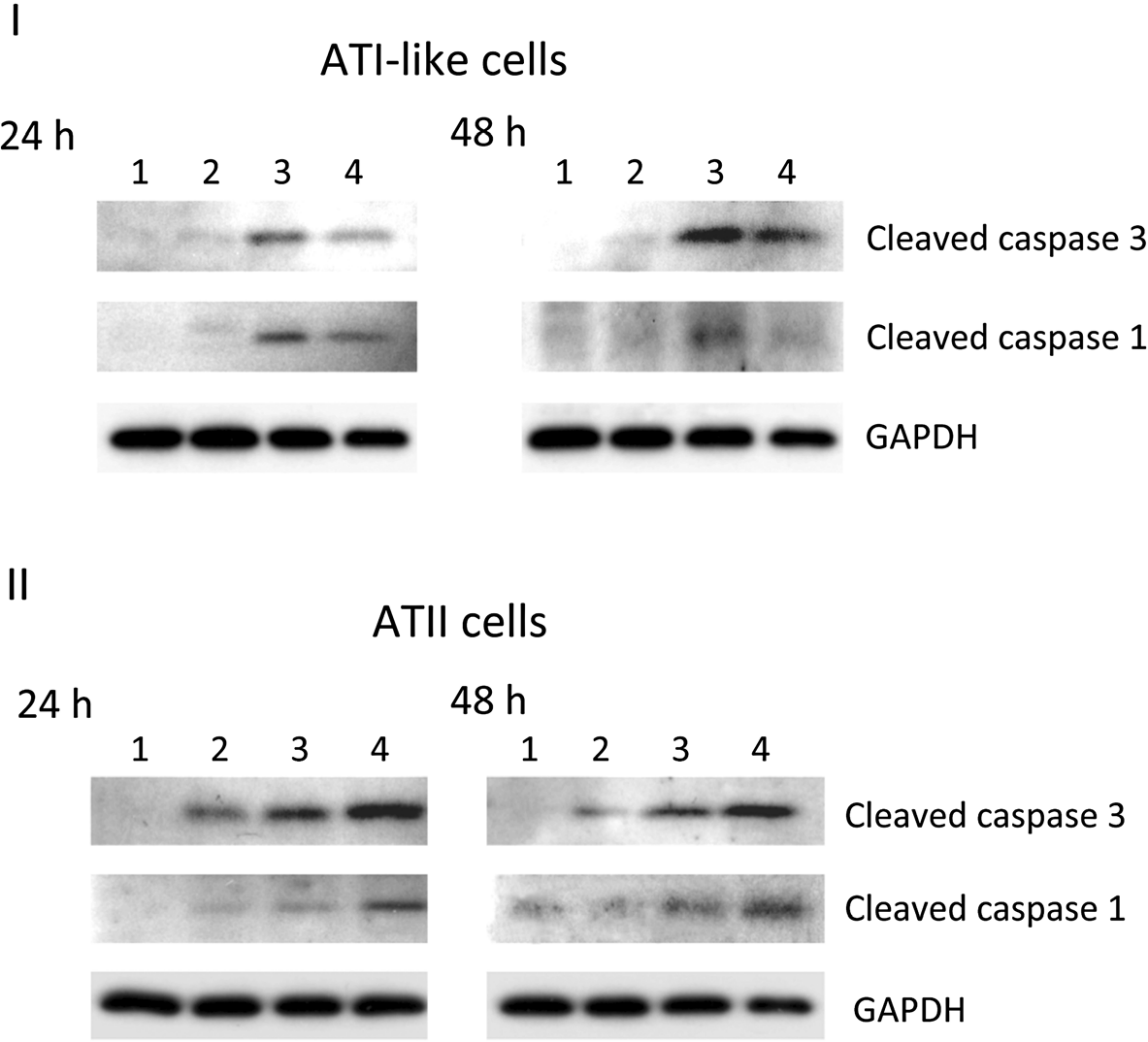
**Caspase 1 and caspase 3 cleavage induced by PR8 virus in ATI-like and ATII cells.** Cells were cultured as described in the Method section and infected at a MOI of 0.05, 0.5 and 1 pfu/cell PR8 virus for 24 h or 48 h. Cleaved caspase 1 and 3 were detected in ATI-like cells (Panel **I**) and in ATII cells (Panel **II**) by immunoblotting. Lane 1 – control, Lane 2 – MOI of 0.05 pfu/cell, Lane 3 – MOI of 0.5 pfu/cell, Lane 4 – MOI of 1 pfu/cell. Representative pictures from three independent experiments.

Subsequently, we wanted to document that apoptosis induced by PR8 virus in alveolar cells is associated with caspase activation. We observed caspase 1 and caspase 3 cleavage (Figure 2) and also PARP cleavage (data not shown) in a concentration-dependent manner after ATI-like and ATII cell infection with PR8 virus at 24 hpi and 48 hpi. Hence, these results indicate that PR8 virus induces apoptosis and necrosis.

### Alveolar macrophages, but not epithelial cells ingest apoptotic viral infected ATII cells

One of the major questions related to apoptosis is what happens to the viral-induced apoptotic cells. Virus-infected cells undergo apoptosis and ingestion of apoptotic cells leads to inhibition of virus spread *in vivo*[36]. When epithelial cells undergo apoptosis, the non-apoptotic epithelial cells actively extrude the apoptotic cells from the monolayer in an actin-dependent process [11,12].

**Figure 3:**
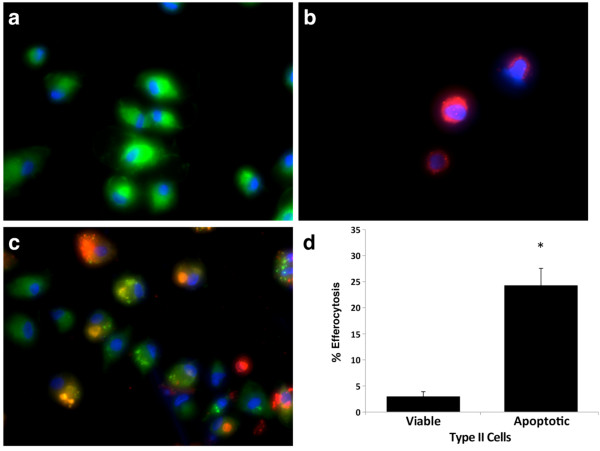
**AM ingest PR8 virus-induced apoptotic ATII cells.** ATII cells were infected with IAV and floating apoptotic cells were collected after 48 h. Floating cells (red) were separately fluorescently stained and added to labeled AM (green) as described in the Methods section. **a** – AM alone; **b** – apoptotic floating ATII cells induced by a MOI of 1 pfu/cell PR8 virus after 48 h; **c** – ingestion of PR8 virus-induced apoptotic ATII cells by AM; **d** – percent of efferocytosis of viable ATII cells and PR8 virus-induced apoptotic ATII cells by AM. Data represent results from three independent experiments (Mean ± SEM, * *p* < 0.05).

We wanted to determine potential clearance routes for the apoptotic cells [10]. We analyzed uptake of viral infected apoptotic or viable ATII cells by AM or ATII cells. We found significant ingestion of apoptotic ATII cells by AM (Figure 3). In addition, under these same conditions ATII cells did not ingest apoptotic ATII cells (data not shown). Removal of apoptotic cells by AM avoids secondary necrosis and the release of cell contents that may promote further inflammation [37]. Our observation that AM ingest influenza A virus-induced apoptotic cells may further improve strategies aimed on the increasing efferocytosis by these cells that may prevent excessive inflammation and pneumonia.

### PR8 virus induces Nrf2 and HO-1 expression and translocation to the nucleus

**Figure 4:**
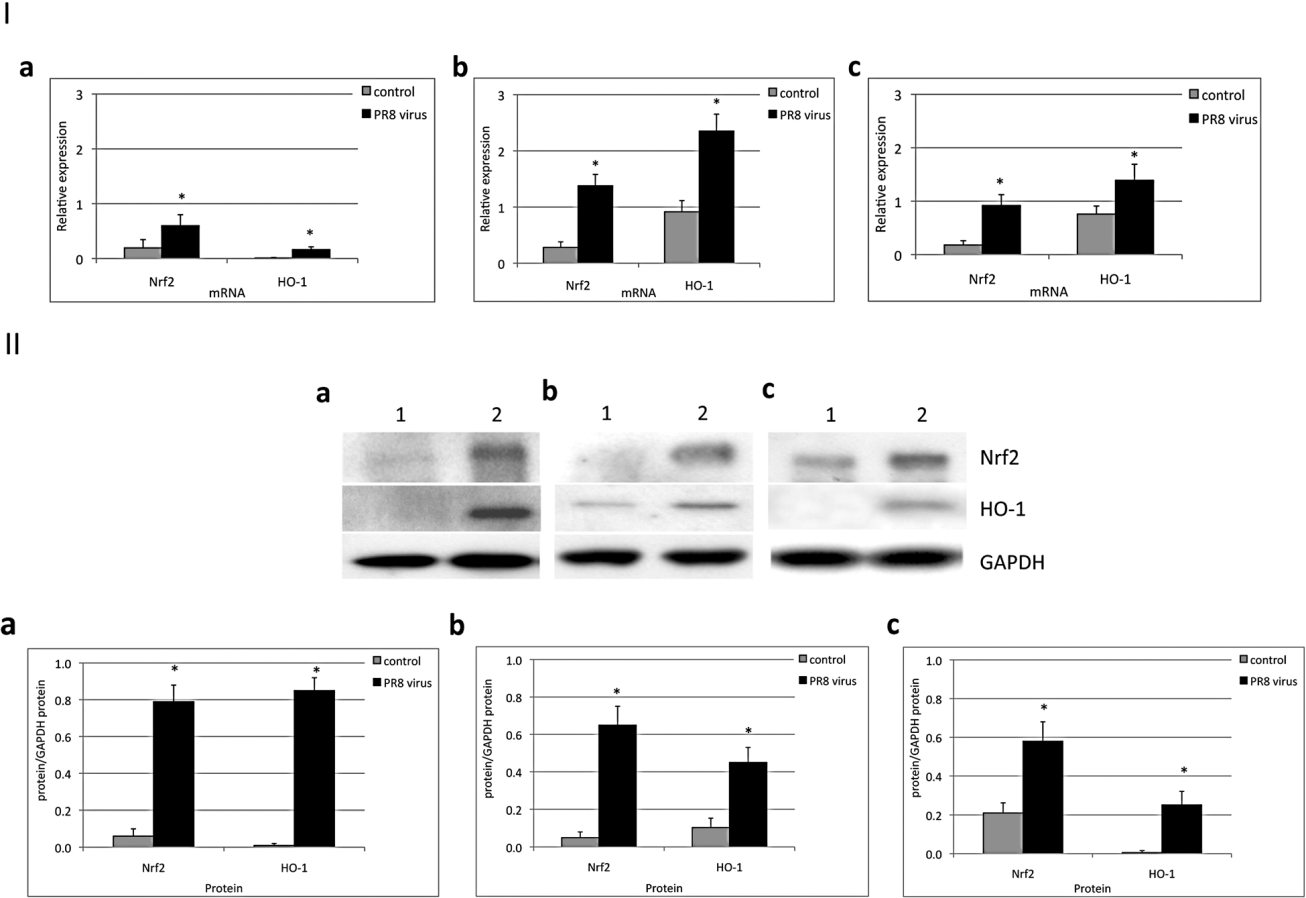
**PR8 virus induces Nrf2 and HO-1 expression.** ATI-like cells (**a**), ATII cells (**b**) and AM (**c**) were grown as described in the Methods section, infected at a MOI of 0.5 pfu/cell PR8 virus for 48 h. Panel **I**, relative expression of Nrf2 and HO-1 as detected by RT-PCR. Panel **II**, Nrf2 and HO-1 expression in alveolar cells (immunoblotting). Lane 1 – control; lane 2 – MOI of 0.5 pfu/cell PR8 virus. Relative expression of these proteins is also shown. Data represent results from three independent experiments (Mean ± SEM, * *p* < 0.05).

We found that PR8 virus at a MOI of 0.5 pfu/cell significantly increased Nrf2 and downstream HO-1 mRNA levels in ATI-like cells, ATII cells and AM after 48 h (Figure 4, Panel I). We also verified these results on the protein level and observed by immunoblotting higher Nrf2 and HO-1 expression in these cells after treatment with IAV (Figure 4, Panel II).

**Figure 5:**
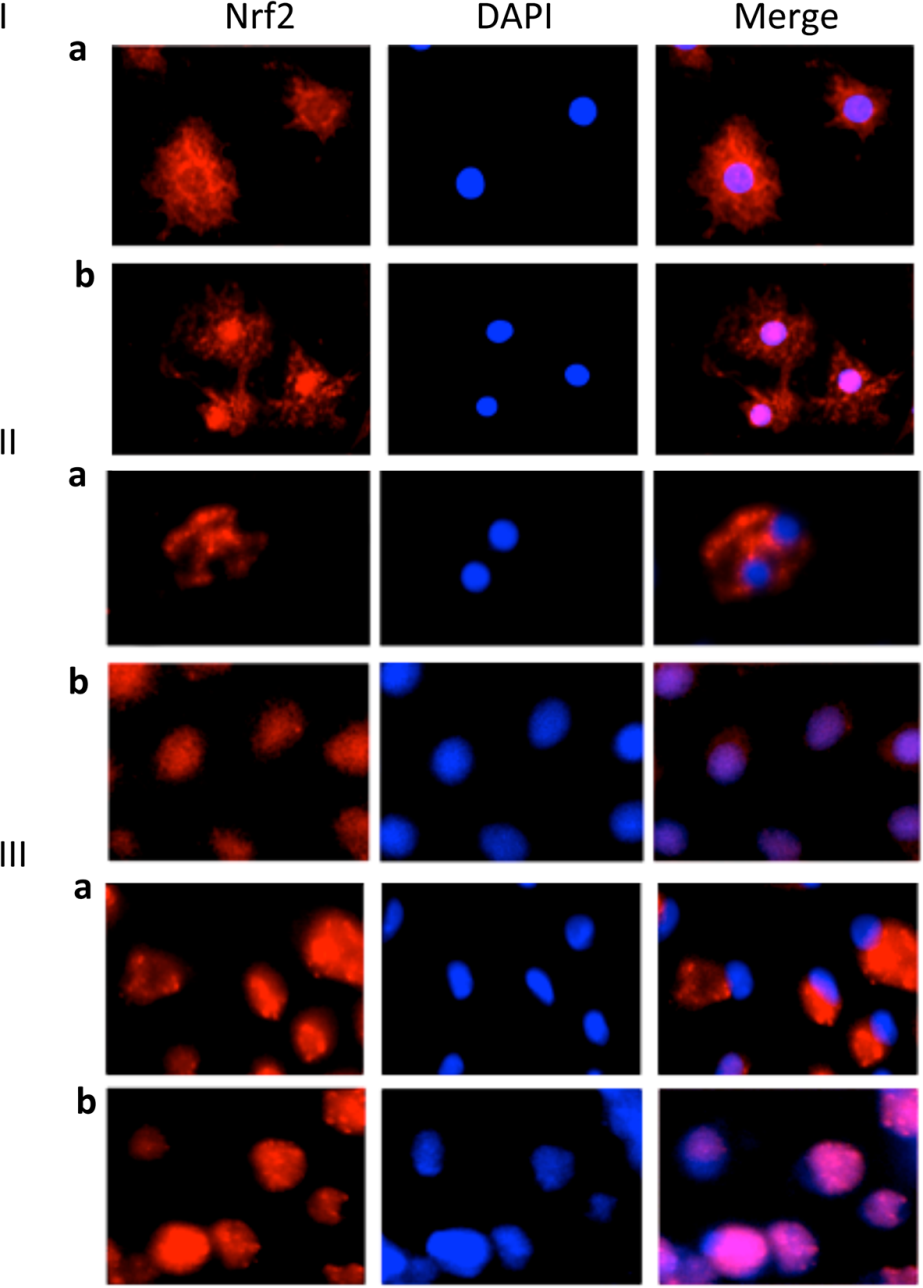
**Nrf2 translocation to the nucleus.** ATI-like cells (Panel **I**), ATII cells (Panel **II**) and AM (Panel **III**) were infected at a MOI of 0.5 pfu/cell PR8 virus and Nrf2 activation was observed by its translocation to the nucleus. **a** – Cytoplasmic localization of Nrf2 in control cells; **b** - Nuclear translocation of Nrf2 after infection with PR8 virus (immunocytofluorescence). Representative pictures from three independent experiments.

We also found Nrf2 translocation to the nucleus in ATI-like cells, ATII cells and AM infected at a MOI of 0.5 pfu/cell PR8 virus for 48 h in comparison with controls (Figure 5). Our results indicate induction of the Nrf2 pathway in these cells in response to IAV infection.

### Nrf2 overexpression protects ATI-like cells against injury induced by PR8 virus

**Figure 6:**
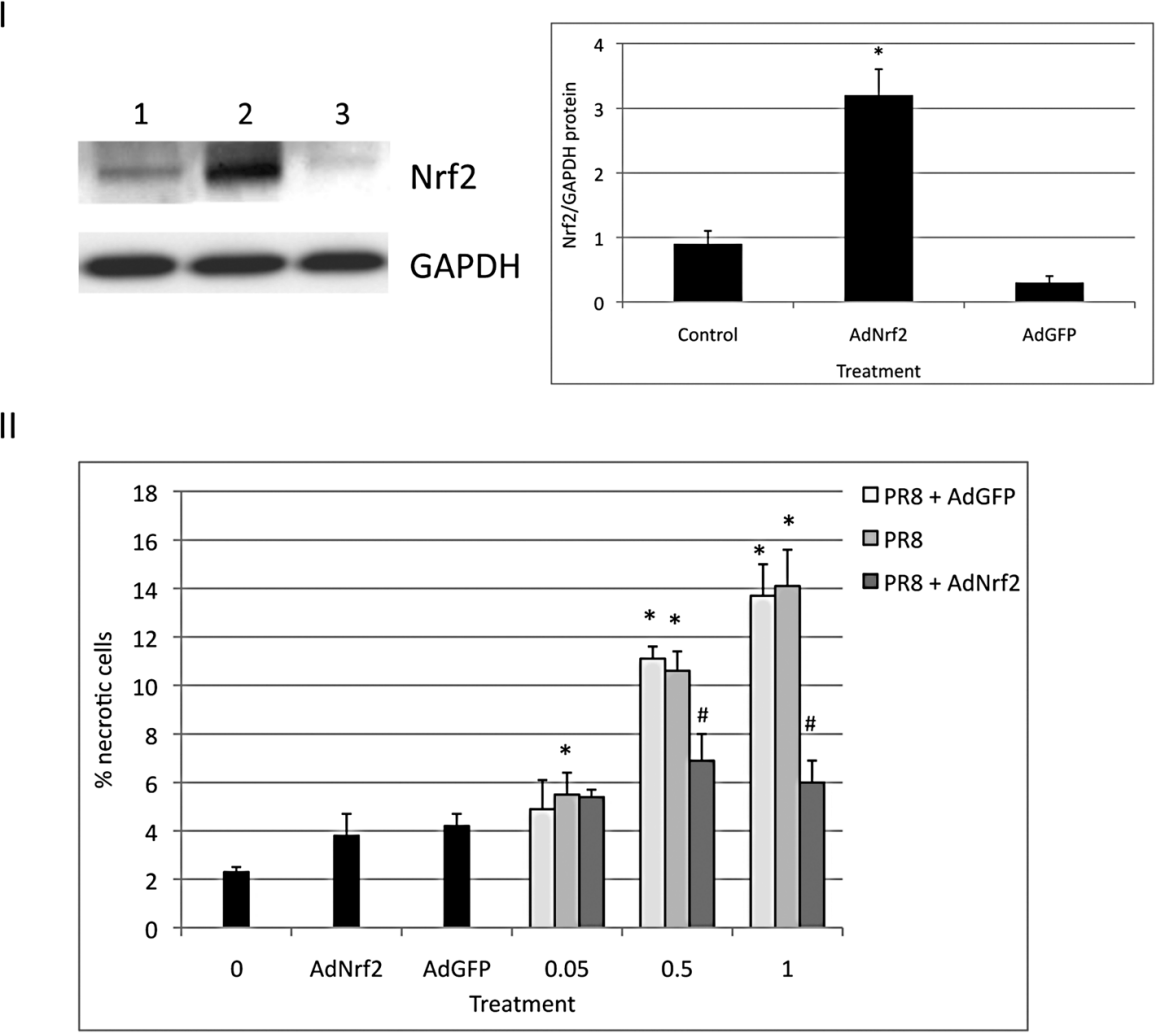
**AdNrf2 protects ATI-like cells against injury by PR8 virus.** ATI-like cells were infected for 24 h at a MOI of 200 pfu/cell AdNrf2 or AdGFP as described in the Methods section. Panel **I** – Nrf2 was overexpressed by AdNrf2 and cells were harvested at 24 hpi: Lane 1 - control; lane 2 - AdNrf2, lane 3 - AdGFP (immunoblotting). Relative expression of this protein is also shown. Panel **II** – Cells were infected with AdNrf2 followed by infection at a MOI of 0.05, 0.5 and 1 pfu/cell IAV for 24 h. A higher percentage of necrotic cells was observed in cells infected with PR8 virus in comparison with cells infected with AdNrf2 and followed by infection with PR8 as detected by Hoechst 33342 and propidium iodide double staining. * Statistically significant increase in the percentage of necrotic cells by PR8 infection in comparison with control. # Statistically significant decrease of ATI-like necrotic cells after infection with AdNrf2 and PR8 in comparison to PR8 virus alone (Mean ± SEM, N = 3, *p* < 0.05). Data represent results from three independent experiments.

Nrf2 translocation to the nucleus indicates activation of the antioxidant defense system mediated by Nrf2. To further study the role of the Nrf2 pathway, we overexpressed Nrf2 in ATI-like cells (Figure 6, Panel I). We infected ATI-like cells with AdNrf2 followed by PR8 virus (Figure 6, Panel II). We found a significantly higher percentage of necrotic cells after cell infection with PR8 virus alone. The highest percentage of necrotic cells (14.1%) was observed at a MOI of 1 pfu/cell. Furthermore, we observed a significant decrease in the percentage of necrotic cells after ATI-like cell infection with AdNrf2 followed by a MOI of 0.5 or 1 pfu/cell PR8 virus in comparison with PR8 virus alone.

**Figure 7:**
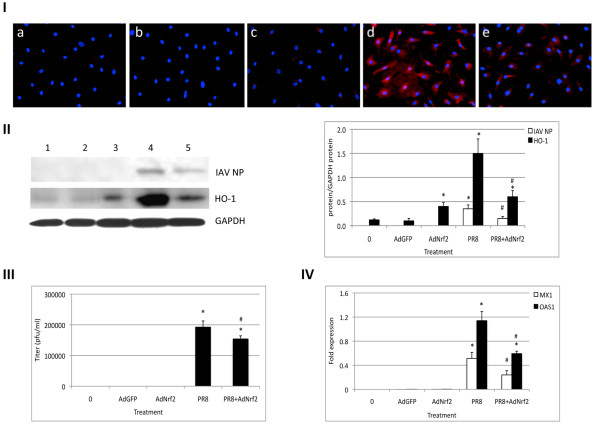
**AdNrf2 decreases IAV infection in ATI-like cells.** Cells were infected at a MOI of 200 pfu/cell AdNrf2 followed by infection at a MOI of 1 pfu/cell PR8 virus for 24 h. Panel **I** – Oxidative stress was measured by immunocytofluorescence with an antibody to 4-HNE as described in the Methods section. **a** - control; **b** – AdGFP, **c** - AdNrf2; **d** – PR8 virus; **e** - AdNrf2 and PR8 virus. Panel **II** – Infection with PR8 virus significantly increased influenza A nucleoprotein and HO-1 expression. Infection with AdNrf2 followed by PR8 virus decreased their expression (immunoblotting). Lane 1- control; lane 2 – AdGFP; lane 3 - AdNrf2; lane 4 – PR8 virus; and lane 5 - AdNrf2 and PR8 virus. Relative expression of these proteins is also shown. Panel **III** – AdNrf2 decreased replication of PR8 virus in comparison with PR8 alone. Cells were infected with IAV or AdNrf2 followed by infection with PR8 virus. Media were harvested 24 h after inoculation and titrated by plaque assay in MDCK cells as described in the Methods section. Panel **IV** – IAV increases antiviral gene Mx1 and OAS1 mRNA levels as measured by RT-PCR. Infection with AdNrf2 followed by PR8 virus significantly decreased their expression. * Statistically significant increase in the gene expression in comparison with control. # Statistically significant decrease in comparison with PR8 virus alone (Mean ± SEM, N = 3, *p* < 0.05).

To determine the potential mechanisms mediating the decreased cell injury after PR8 infection due to Nrf2 overexpression, we assessed oxidative stress. To do this we analyzed the level of 4-HNE. PR8 virus induced oxidative stress and 4-HNE immunostaining was decreased after cell infection with AdNrf2 followed by PR8 virus (Figure 7, Panel I). To further investigate the mechanisms responsible for ATI-like cell protection after Nrf2 overexpression, we evaluated the expression of HO-1, which is a well-defined target of Nrf2 and induced by oxidative stress (Figure 7, Panel II). We found that PR8 virus significantly increases HO-1 level, and this expression was decreased after infection with AdNrf2 followed by PR8 virus. These results suggest that infection of ATI-like cells with PR8 virus induces oxidative stress, which was diminished by Nrf2 overexpression.

Next we wanted to determine whether AdNrf2 affects IAV replication. Culture media from ATI-like cells inoculated with PR8 virus were collected and titrated by plaque assay. We found that infection with AdNrf2 followed by IAV slightly decreased PR8 virus titer released into the media (Figure 7, Panel III). We also observed a significant increase in influenza A nucleoprotein expression in ATI-like cells infected with PR8 virus at a MOI of 1 pfu/cell and a slight decrease after infection with AdNrf2 followed by IAV (Figure 7, Panel II). These results suggest that Nrf2 overexpression decreased viral replication in ATI-like cells.

To determine the potential mechanisms mediating decreased IAV replication after infection with AdNrf2, we assessed antiviral immune response mediators. Specifically, we analyzed the effect of AdNrf2 alone or Nrf2 overexpression followed by infection with PR8 virus on the interferon-induced Mx1 and the OAS1 genes, which are involved in the innate immune response to viral infection [25]. Nrf2 overexpression did not increase Mx1 and OAS1 mRNA (Figure 7, Panel IV). However, infection with AdNrf2 followed by PR8 virus significantly reduced expression of these genes in comparison with IAV alone which indicates cytoprotective mechanisms orchestrated by Nrf2 against cell injury by IAV.

In summary, our data demonstrate that expression level of Nrf2 plays a role in decreasing infection of IAV in ATI-like cells by antiviral activity of Nrf2, reducing oxidative stress and induction of cellular defense systems.

### Nrf2 overexpression increases IL-8 but not IL-29 secretion

**Figure 8:**
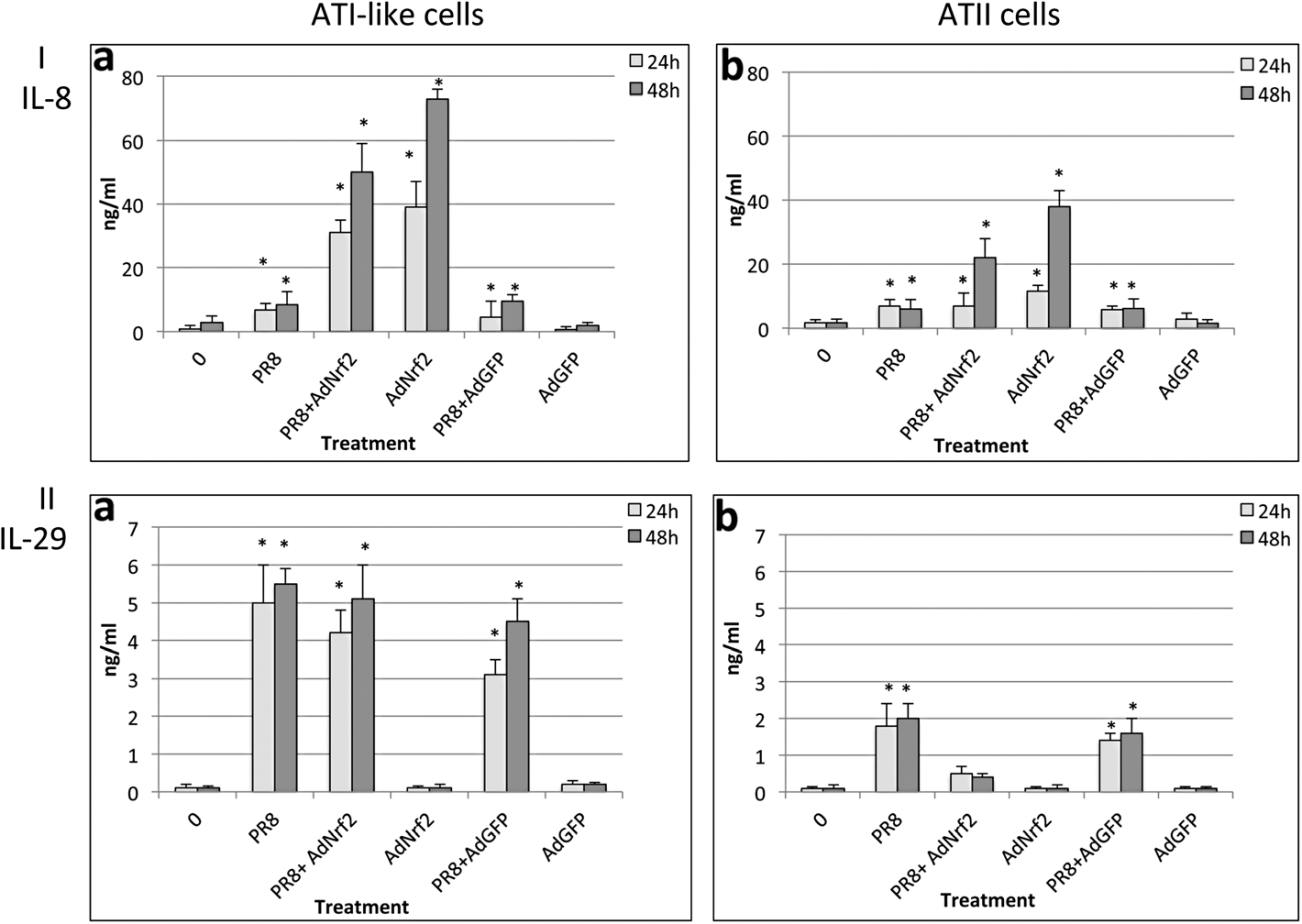
**AdNrf2 increases IL-8 secretion but does not alter the IL-29 response.** IL-8 (Panel **I**) and IL-29 (Panel **II**) were analyzed in ATI-like (**a**) and ATII (**b**) cells by ELISA. ATI-like or ATII cells were cultured as described in the Methods section. Cells were infected at a MOI of 1 pfu/cell PR8 virus, infected with AdNrf2 or AdGFP for 24 h and/or followed by infection with PR8 for 24 h or 48 h. * Statistically significant increase in cytokine secretion in comparison with control (Mean ± SEM, *p* < 0.05). Data represent results from three independent experiments.

To further investigate the mechanism of Nrf2 protection against IAV we analyzed cytokine levels. PR8 virus significantly increased IL-8 secretion in comparison with untreated controls (Figure 8, Panel I). Unexpectedly, we observed a much higher IL-8 secretion by AdNrf2 in comparison with cells infected with AdGFP in ATI-like (Figure 8, Panel I, a) and ATII cells (Figure 8, Panel I, b). We also found that PR8 virus significantly increased IL-29 secretion in ATI-like cells (Figure 8, Panel II, a) and ATII cells (Figure 8, Panel II, b). IAV induced higher IL-29 expression in ATI-like than ATII cells. However, we did not observe higher IL-29 levels after cell infection with AdNrf2 or AdGFP. These results confirm our previous observations that the interferon response to PR8 virus is not altered by Nrf2 levels.

### Nrf2 knockdown sensitizes cells to injury induced by PR8 virus

**Figure 9:**
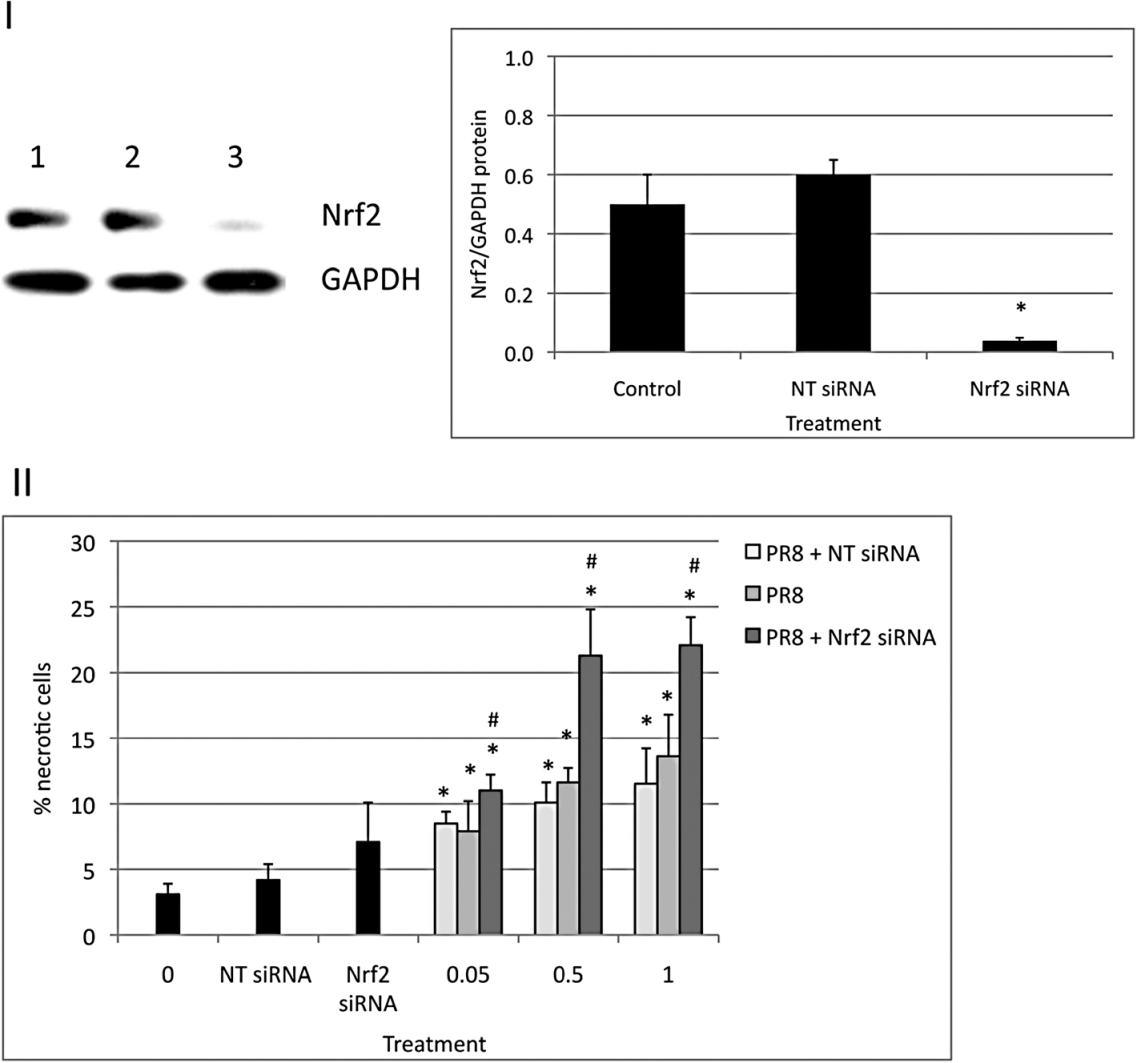
**Nrf2 knockdown sensitizes ATI-like cells to PR8 virus.** ATI-like cells were transfected with 100 nmol Nrf2 siRNA or NT siRNA for 24 h as described in the Methods section. Panel **I** – Nrf2 was knocked down by Nrf2 siRNA in cells harvested at 24 hpi: Lane 1 – control; lane 2 - NT siRNA; lane 3 - Nrf2 siRNA (immunoblotting). Relative expression of these proteins is also shown. Panel **II** – A higher percentage of necrotic cells was observed in cells transfected with Nrf2 siRNA for 24 h and followed by infection with PR8 virus for 24 h in comparison with cells infected with PR8 virus alone. * Statistically significant increase in percentage of necrotic cells induced by PR8 virus in comparison with control. # Statistically significant increase of ATI-like necrotic cells after transfection with Nrf2 siRNA followed by PR8 in comparison with PR8 virus alone. Data represent results from three independent experiments (Mean ± SEM, * *p* < 0.05).

To verify our results of the protective role of the Nrf2 pathway in cells infected with PR8 virus, we knocked down Nrf2 and then infected cells with PR8 virus. We were able to knockdown Nrf2 in ATI-like cells using Nrf2 siRNA (Figure 9, Panel I). We found that Nrf2 knockdown followed by infection with PR8 virus increased the cytotoxicity for all applied virus concentrations in comparison with PR8 virus alone (Figure 9, Panel II). This indicates that Nrf2 knockdown sensitizes ATI-like cells to injury induced by PR8 virus and Nrf2 level modulates cell injury by IAV.

### PR8 virus generates ROS

**Figure 10:**
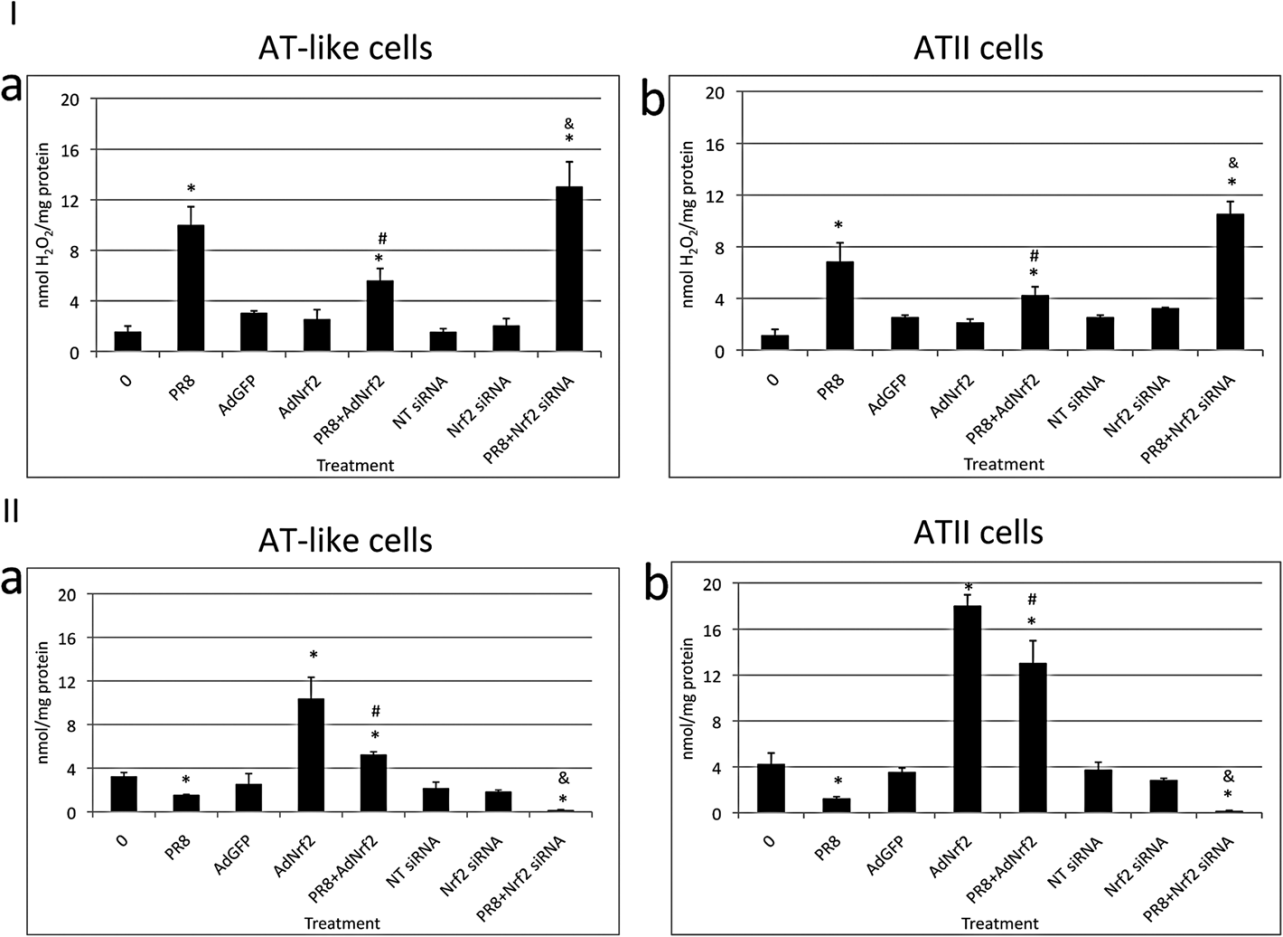
**AdNrf2 protects cells and Nrf2 siRNA sensitizes cells to injury induced by PR8 virus.** ATI-like cells (**a**) and ATII cells (**b**) were grown as described in the Methods section and infected with PR8 virus at a MOI of 0.5 pfu/cell for 24 h. Panel **I**, cell infection with AdNrf2 followed by infection with PR8 virus decreased ROS generation in comparison with PR8 alone as measured by Amplex Red kit. Cell transfection with Nrf2 siRNA followed by infection with PR8 virus increased ROS production in comparison with PR8 alone. * Statistically significant difference in comparison with control. # Statistically significant decrease in comparison with PR8 virus alone. & - Statistically significant increase in comparison with PR8 virus alone. Data represent results from three independent experiments. Panel **II**, AdNrf2 increased GSH level in comparison with control. Cell transfection with Nrf2 siRNA followed by infection with PR8 virus decreased GSH level in comparison with PR8 virus alone. * Statistically significant difference in comparison with control. # Statistically significant increase in comparison with PR8 virus alone. & - Statistically significant decrease in comparison with PR8 virus alone. Data represent results from three independent experiments (Mean ± SEM, * *p* < 0.05).

Among the methods commonly used to measure H_2_O_2_, AmplexRed has several advantages. The ability to simply and accurately calibrate signals to peroxide concentrations offers the opportunity to carefully quantitate the production of oxidants by biological systems [33]. We transfected ATI-like cells and ATII cells with Nrf2 siRNA or AdNrf2 followed by infection at a MOI of 0.5 pfu/cell PR8 virus for 24 h. We found significantly higher ROS generation in cells transfected with Nrf2 siRNA followed by infection with PR8 virus in comparison with IAV alone (Figure 10, Panel I). To complete our results we also infected these cells with AdNrf2 followed by PR8 virus and we found lower ROS generation in comparison with IAV alone. Our results indicate the protective role of Nrf2 against ROS generation by PR8 virus and are in agreement with a positive staining using 4-HNE (Figure 7).

### AdNrf2 increases glutathione level

To determine the protective mechanism of Nrf2 against oxidative stress induced by IAV we measured GSH level in ATI-like and ATII cells transfected with Nrf2 siRNA or AdNrf2 followed by infection at a MOI of 0.5 pfu/cell PR8 virus for 24 h. We found significantly lower levels of GSH after cell tranfection with Nrf2 siRNA followed by infection with PR8 virus (Figure 10, Panel II). Moreover, AdNrf2 increased GSH levels, which protected cells against injury by IAV. These results indicate that the protective role of Nrf2 against cell injury induced by PR8 virus is in part by increasing glutathione levels.

## Discussion

To date, whether or to what extent oxidative stress contributes to the highly virulent property of influenza virus is not fully known. We observed that PR8 virus induced oxidative stress, cell injury, apoptosis, and proinflammatory cytokine secretion in ATI-like cells and ATII cells. In order to protect cells from injury induced by the PR8 virus, host cells must activate some defense against oxidative stress. Nrf2 is critical factor and can activate antioxidant response genes. Ours is a novel approach in that first, we studied the response of ATI-like cells infected with IAV, second, compared the effect of PR8 virus on ATI-like cells, ATII cells and AM obtained from the same donors and third, to our knowledge this is the first report on the role of Nrf2 in alveolar cells infected with influenza A virus.

Our results clearly show that Nrf2 regulates alveolar cells’ susceptibility to infection and injury induced by PR8 virus. We confirmed our hypotheses and demonstrated the protective role of Nrf2 against PR8 virus infection. To our knowledge there are only two reports on the role of Nrf2 and influenza virus. These studies were performed in human nasal epithelial cells *in vitro*[5] and in mice *in vivo*[6]. Cigarette smoke-exposed Nrf2^-^/^-^ mice showed higher rates of mortality than did wild-type mice after IAV infection, with higher peribronchial inflammation, lung permeability damage, and mucus hypersecretion. It has been recently reported that supplementation with the potent Nrf2 activator epigallocatechin gallate significantly decreased influenza A/Bangkok/1/79 virus entry and replication in nasal epithelial cells [5]. The suppressive effect of this compound on viral replication was abolished in cells with knocked down Nrf2 expression. This suggests a relationship between induction of the Nrf2 pathway and the ability to protect against viral infection. Our results are in agreement with these findings. We found Nrf2 translocated to the nucleus in ATI-like cells, ATII cells and AM infected with PR8 virus. We also showed significant induction of Nrf2 and downstream HO-1. This suggests activation of the Nrf2 pathway in response to IAV infection. We also observed the protective effect of Nrf2 against IAV in human alveolar cells. We found that Nrf2 overexpression using AdNrf2 decreased IAV replication, influenza A nucleoprotein expression, antiviral gene expression, ROS generation and oxidative stress in comparison with PR8 virus alone. Furthermore, AdNrf2 also increased GSH level in ATI-like and ATII cells, which protected against injury by PR8 virus. Shih et al. [27] also observed higher intracellular GSH levels in neurons infected with AdNrf2, which protected against oxidative stress and GSH has been reported to be an inhibitor of IAV infection [38]. Moreover, we found that Nrf2 knockdown followed by infection with PR8 virus decreased cell viability, increased ROS generation and decreased GSH levels. Kesic et al. [5] observed that epigallocatechin gallate increased expression of antiviral genes RIG-1, IFN-β and Mx1. However, these authors suggested that their expression can be Nrf2-independent or that the effects are species and/or cell-type specific. We found that AdNrf2 did not induce mRNA levels of the antiviral genes Mx1 and OAS1. Our results can be explained by a different strategy to overexpress Nrf2 and/or the cell type studied. Moreover, to our knowledge, essential Nrf2 binding sites have not been identified in promoters of any of these genes [5]. Our results are also in agreement with studies of gene expression profiling Nrf2 in mice showing that there are no differences in the antiviral or interferon responsive genes [5,39,40]. Our results suggest that the activation of antioxidant and cytoprotective mechanisms orchestrated by Nrf2 (e.g., HO-1 activation) are responsible for cell protection against IAV. To our knowledge, this is the first observation on the antiviral role of Nrf2 in human alveolar epithelial cells.

We found that MOI of 1 pfu/cell PR8 virus induces IL-8 and IL-29 secretion in ATI-like and ATII cells. Surprisingly, we observed that AdNrf2 increased IL-8 levels but not IL-29. It was postulated that the Nrf2/antioxidant response pathway regulates IL-8 expression and Nrf2-dependent RNA binding protein may directly stabilize IL-8 mRNA [41,42]. The protective effect of Nrf2 also suggests that under certain circumstances, IL-8 might have a protective function and serves an anti-inflammatory role in remodeling during the resolution of inflammation.

Apoptosis induced by influenza virus has been shown in a variety of cell lines [43-45]. We observed both necrosis and apoptosis induced by PR8 virus in primary human ATI-like and ATII cells. We found chromatin fragmentation and condensation in attached cells and floating apoptotic cells after infection with PR8 as detected by TUNEL assay. Floating murine primary apoptotic macrophages were observed after treatment with PR8 virus [46]. Furthermore, Eckardt-Michel et al. [47] found that the fusion protein of RSV also induced floating cells that have characteristics of the apoptotic DNA ladder, which suggests that they are extruded from the monolayer before late apoptotic events became apparent. In our studies these floating viral infected apoptotic cells were ingested by AM but not by ATII cells. These results suggest that inflammatory macrophages not the resident epithelial cells are likely responsible for clearing IAV-induced apoptotic cells. This is different from the involutional mammary gland where the epithelium itself is responsible for clearing the apoptotic epithelial cells [10,11].

To investigate the characteristics of the onset of apoptosis ATI-like and ATII cells infected with PR8 virus, we measured caspase 1 and 3 cleavage. Our results are in agreement with our previous study showing caspase 3 and PARP cleavage in ATII cells isolated from adult lung donors and infected at a MOI of 0.5 pfu/cell PR8 for 24 h [19]. This is the first study on the effect of IAV on ATI-like cells and the results are in agreement with studies showing involvement of caspases in apoptosis upon influenza virus infection in cell lines and human monocyte-derived macrophages [43,48,49].

In summary, the effects of Nrf2 activation during influenza infections are complex. There is inhibition of viral replication, which might be due to reduced viral entry [5]. There is apparently no alteration in the anti-viral genes examined or in the interferon response. There is a significant antioxidant pro-survival response typical for the Nrf2 pathway in oxidative stress.

## Conclusions

We used ATI-like, ATII cells and AM to study a response to IAV and to show for the first time, the protective role of Nrf2 in human alveolar cells. Our results suggest that Nrf2 is involved in the cellular antioxidant defense system, is activated upon infection with PR8 virus, and protects the host from the cytopathic effects of oxidative stress induced by IAV in interferon-independent manner. Taken together, our results indicate that Nrf2 is an important factor that can modify the response to PR8 virus. Identifying the pathways involved in the cell response to this infection are particularly important for new therapeutic strategies. Nevertheless, this study will need to be compared with cells from other vulnerable populations, such as cigarette smokers, and patients with chronic obstructive pulmonary disease. Additional studies will be necessary to fully understand the role of Nrf2 in the pathogenesis of viral pneumonia (Additional file 3).

## Abbreviations

(ATII cells): Alveolar type II cells; (ATI-like cells): Alveolar type I-like cells; (AM): Alveolar macrophage; (Nrf2): Nuclear factor-erythroid 2 related factor 2; (AdNrf2): Adenovirus Nrf2; (AdGFP): Adenovirus GFP; (PR8): A/PR/8/34; (IAV): Influenza A virus; (HO-1): Heme oxygenase-1; (IL-8): Interleukin 8; (IL-29): Interferon-λ1; (4-HNE): 4-hydroxynonenal; (TUNEL): TdT-mediated dUTP Nick-End Labeling assay; OAS1: 2^′^,5^′^-oligoadenylate synthetase 1.

## Competing interests

The authors declare that they have no competing interests.

## Authors’ contribution

Conceived and designed the experiments: BK, RJM. Performed the experiments: BK, EM, PN, WJJ, JW. Analyzed the experiments: BK, EM, RJM. Contributed reagents/Materials: KLH. Wrote the paper: BK, RJM. All authors read and approved the manuscript.

## Supplementary Material

Additional file 1 **Figure S1.** ATI-like cells are more sensitive to PR8 virus. ATI-like (Panel I) and ATII (Panel II) cells were infected with PR8 virus at a MOI of 0.05, 0.5 or 1 pfu/cell and cell viability was assessed 24 h and 48 h after cell inoculation. The percent of cells that were injured as measured by Hoechst 33342 and propidium iodide double staining is shown. There was much more injury in the floating cells (b) than the attached cells (a). * - Statistically significant increase in percentage of necrotic cells induced by PR8 virus in comparison with control. # - Statistically significant increase of ATI-like necrotic cells in comparison with necrotic ATII cells after infection with A/PR/8/3 virus. Data represent results from three independent experiments (*p*<0.05). Click here for file

Additional file 2 **Figure S2.** PR8 virus induces apoptosis in ATI-like cells and ATII cells. Representative pictures of apoptotic cells infected with IAV and detected by TUNEL assay. Panel I – Morphological characteristics of apoptosis in ATI-like cells infected at a MOI of 1 pfu/cell PR8 virus and harvested at 48 hpi: A - chromatin condensation in attached apoptotic cells; B – chromatin fragmentation in attached apoptotic cells; C – floating apoptotic cells (cytospin). Green are TUNEL-positive cells. Panel II – Quantation of apoptosis in attached and floating ATI-like (a) and ATII (b) cells infected at a MOI of 0.05, 0.5 and 1 pfu/cell PR8 virus as described in Method section. * Statistically significant increase in percentage of apoptotic cells induced by PR8 virus in comparison with control. # Statistically significant increase of attached or floating ATI-like apoptotic cells in comparison with attached or floating apoptotic ATII cells, respectively after infection with PR8 virus. Data represent results from three independent experiments (*p*<0.05). Click here for file

Additional file 3 This manuscript contains an online supplement with additional method. Click here for file
