# Supplementary figures and images for: Nrf2 protects human alveolar epithelial cells against injury induced by influenza A virus
Source: Respir Res. 2012 Jun 6;13(1):43. doi: 10.1186/1465-9921-13-43 (PMC3520784; doi:10.1186/1465-9921-13-43)

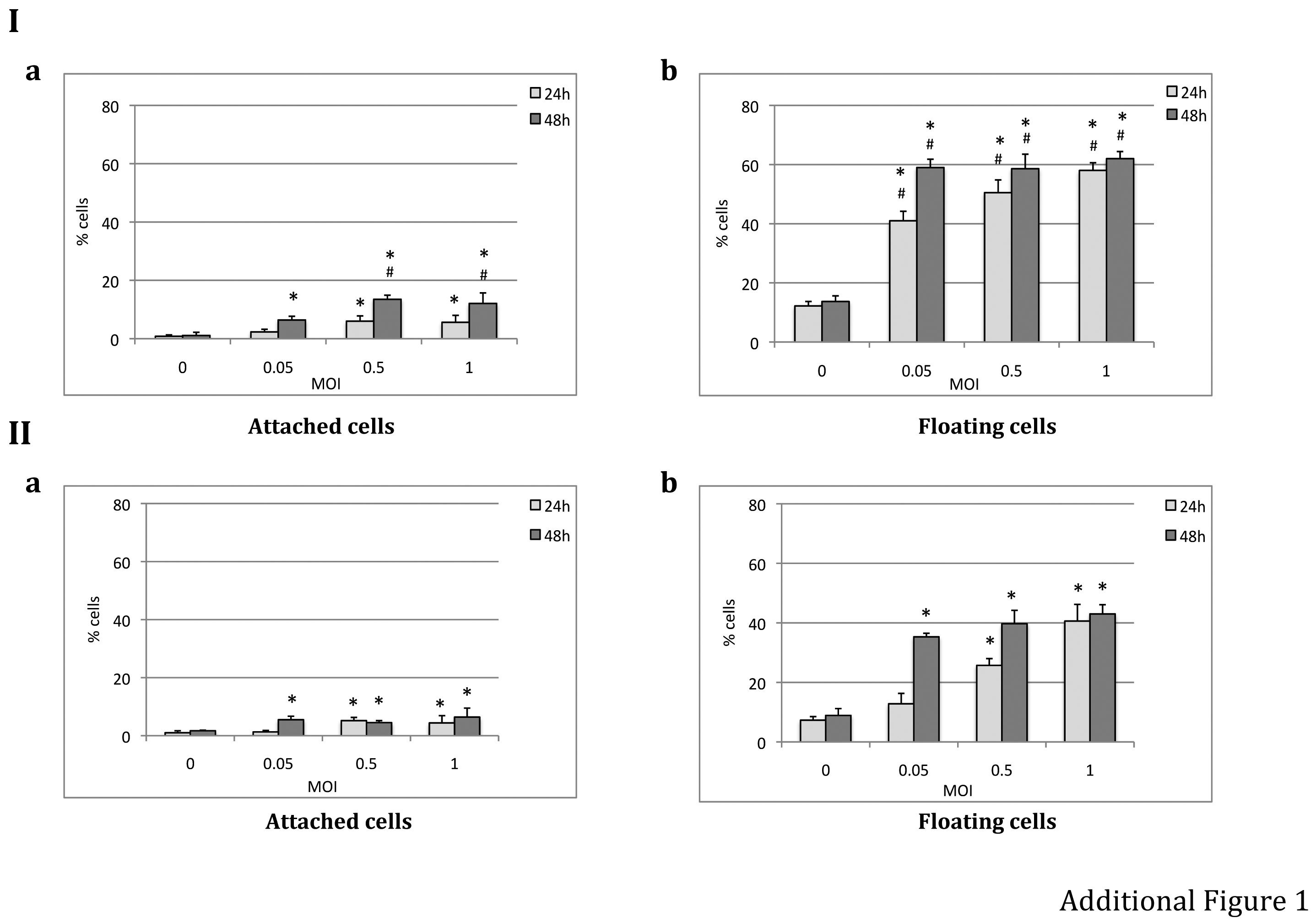

Supplement: Additional file 1 — Figure S1. ATI-like cells are more sensitive to PR8 virus. ATI-like (Panel I) and ATII (Panel II) cells were infected with PR8 virus at a MOI of 0.05, 0.5 or 1 pfu/cell and cell viability was assessed 24 h and 48 h after cell inoculation. The percent of cells that were injured as measured by Hoechst 33342 and propidium iodide double staining is shown. There was much more injury in the floating cells (b) than the attached cells (a). * - Statistically significant increase in percentage of necrotic cells induced by PR8 virus in comparison with control. # - Statistically significant increase of ATI-like necrotic cells in comparison with necrotic ATII cells after infection with A/PR/8/3 virus. Data represent results from three independent experiments (p<0.05). [file 1465-9921-13-43-S1.tiff]

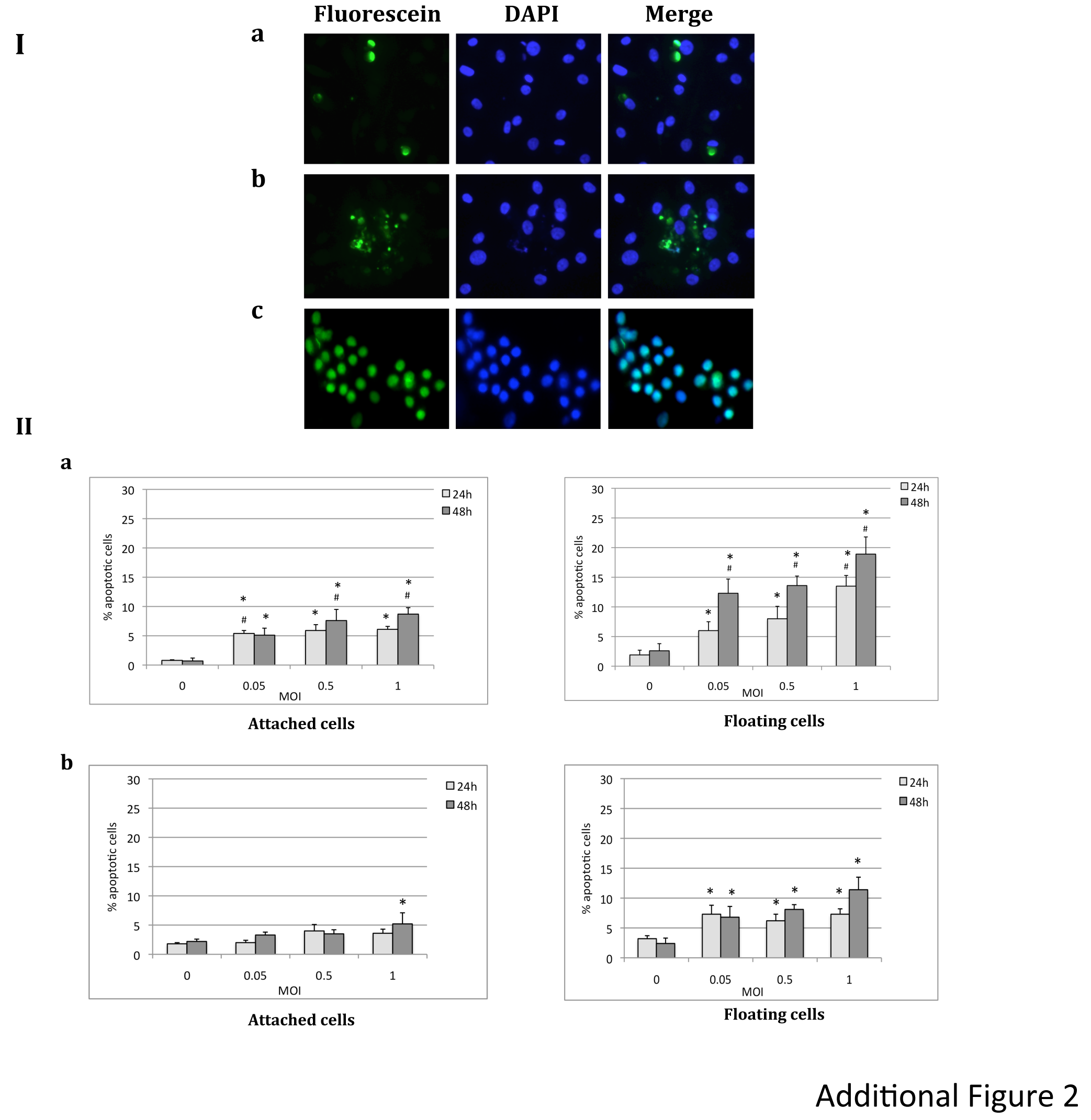

Supplement: Additional file 2 — Figure S2. PR8 virus induces apoptosis in ATI-like cells and ATII cells. Representative pictures of apoptotic cells infected with IAV and detected by TUNEL assay. Panel I – Morphological characteristics of apoptosis in ATI-like cells infected at a MOI of 1 pfu/cell PR8 virus and harvested at 48 hpi: A - chromatin condensation in attached apoptotic cells; B – chromatin fragmentation in attached apoptotic cells; C – floating apoptotic cells (cytospin). Green are TUNEL-positive cells. Panel II – Quantation of apoptosis in attached and floating ATI-like (a) and ATII (b) cells infected at a MOI of 0.05, 0.5 and 1 pfu/cell PR8 virus as described in Method section. * Statistically significant increase in percentage of apoptotic cells induced by PR8 virus in comparison with control. # Statistically significant increase of attached or floating ATI-like apoptotic cells in comparison with attached or floating apoptotic ATII cells, respectively after infection with PR8 virus. Data represent results from three independent experiments (p<0.05). [file 1465-9921-13-43-S2.tiff]
