# Supplementary material for: Nrf2 protects human alveolar epithelial cells against injury induced by influenza A virus
Source: Respir Res. 2012 Jun 6;13(1):43. doi: 10.1186/1465-9921-13-43 (PMC3520784; doi:10.1186/1465-9921-13-43)
Supplement: Additional file 3 — This manuscript contains an online supplement with additional method. [file 1465-9921-13-43-S3.doc]

**Additional Files**

**Nrf2 protects human alveolar epithelial cells against injury induced by**

**influenza A virus**

Beata Kosmider^1^, Elise M. Messier^1^, William J. Janssen^1^, Piruz Nahreini^1^, Jieru Wang^1^, Kevan L. Hartshorn^2^, Robert J. Mason^1^

^1^ Department of Medicine, National Jewish Health, 1400 Jackson Street, Denver, CO 80206

^2^ Department of Hematology/Oncology, Boston University School of Medicine, Boston, MA 02118

**Running Title: Nrf2 protects against influenza A virus**

Correspondence:

Beata Kosmider, Ph.D.

Department of Medicine
National Jewish Health
1400 Jackson Street
Denver, CO 80206

E-mail: [KosmiderB@NJHealth.org](mailto:KosmiderB@NJHealth.org)

Phone: +1 303 270 2036

Fax: + 1 303 270 2353

**Methods**

**TUNEL assay**

The induction of apoptosis in ATI-like cells and ATII cells by PR8 virus was analyzed using the TdT-mediated dUTP Nick-End Labeling (TUNEL; Promega, Madison, WI) assay as reported previously [1]. Cells were mounted with Vectashield medium containing DAPI. The percentage of apoptotic cells labeled with fluorescein (TUNEL-positive) was calculated per 10 high-power ﬁelds [2].

**Legend to additional figures**

**Additional Figure 1. ATI-like cells are more sensitive to PR8 virus.** ATI-like (Panel I) and ATII (Panel II) cells were infected with PR8 virus at a MOI of 0.05, 0.5 or 1 pfu/cell and cell viability was assessed 24 h and 48 h after cell inoculation. The percent of cells that were injured as measured by Hoechst 33342 and propidium iodide double staining is shown. There was much more injury in the floating cells (b) than the attached cells (a). * - Statistically significant increase in percentage of necrotic cells induced by PR8 virus in comparison with control. # - Statistically significant increase of ATI-like necrotic cells in comparison with necrotic ATII cells after infection with A/PR/8/3 virus. Data represent results from three independent experiments (*p*<0.05).

**Additional Figure 2. PR8 virus induces apoptosis in ATI-like cells and ATII cells.** Representative pictures of apoptotic cells infected with IAV and detected by TUNEL assay. Panel I – Morphological characteristics of apoptosis in ATI-like cells infected at a MOI of 1 pfu/cell PR8 virus and harvested at 48 hpi: A - chromatin condensation in attached apoptotic cells; B – chromatin fragmentation in attached apoptotic cells; C – floating apoptotic cells (cytospin). Green are TUNEL-positive cells. Panel II – Quantation of apoptosis in attached and floating ATI-like (a) and ATII (b) cells infected at a MOI of 0.05, 0.5 and 1 pfu/cell PR8 virus as described in Method section. * Statistically significant increase in percentage of apoptotic cells induced by PR8 virus in comparison with control. # Statistically significant increase of attached or floating ATI-like apoptotic cells in comparison with attached or floating apoptotic ATII cells, respectively after infection with PR8 virus. Data represent results from three independent experiments (*p*<0.05).

**References**

1. Kosmider B, Zyner E, Osiecka R, Ochocki J: **Induction of apoptosis and necrosis in A549 cells by the cis-Pt(II) complex of 3-aminoflavone in comparison with cis-DDP**. *Mutat Res* 2004, **563**(1):61-70.

2. Ginzberg HH, Shannon PT, Suzuki T, Hong O, Vachon E, Moraes T, Abreu MT, Cherepanov V, Wang X, Chow CW *et al*: **Leukocyte elastase induces epithelial apoptosis: role of mitochondial permeability changes and Akt**. *Am J Physiol Gastrointest Liver Physiol* 2004, **287**(1):G286-298.
